# Supplementary figures and images for: Reassessing the role of the Escherichia coli CpxAR system in sensing surface contact
Source: PLoS One. 2018 Nov 9;13(11):e0207181. doi: 10.1371/journal.pone.0207181 (PMC6226299; doi:10.1371/journal.pone.0207181)

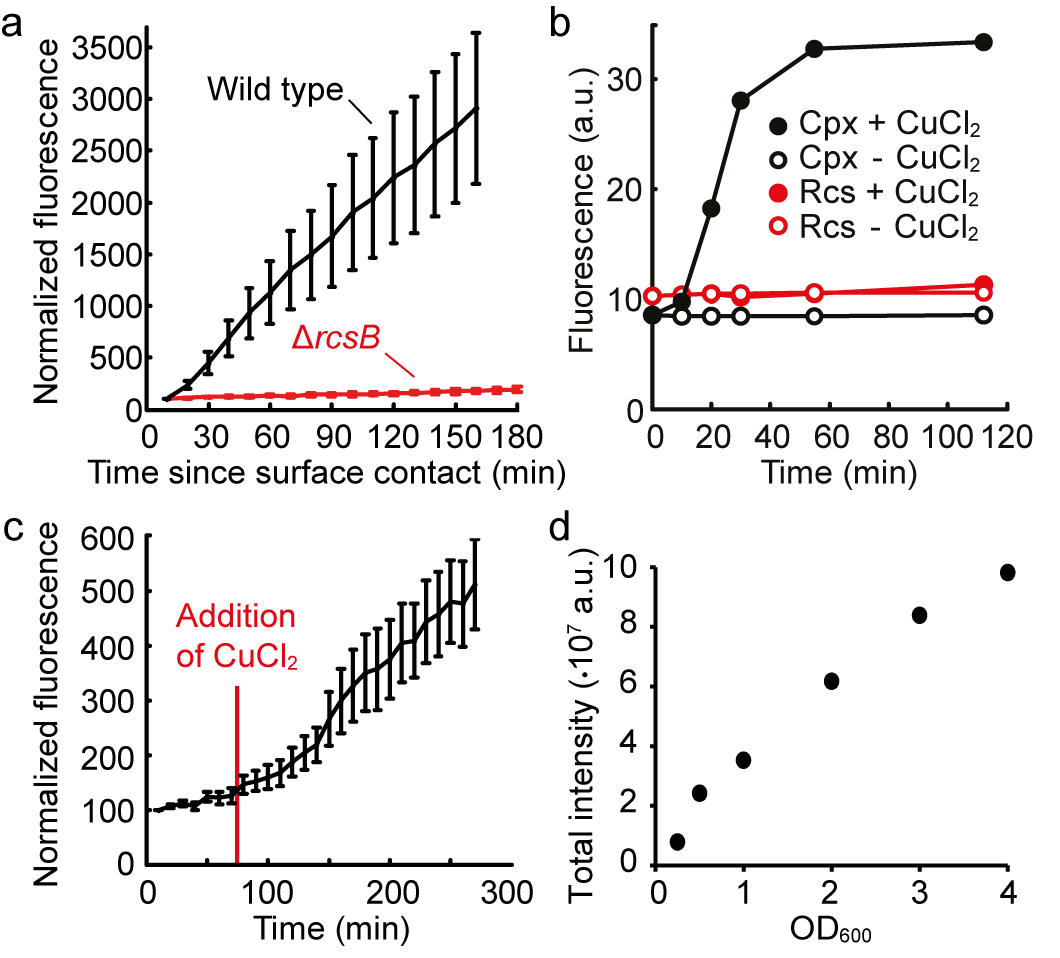

Supplement: S1 Fig — (a) Comparison of GFP expression from the Rcs-regulated rcsA promoter in wild type (black; n = 46; 2 independent experiments) and ΔrcsB cells (red; n = 40; 2 independent experiments) on untreated cover glasses, in the microfluidic device with flow of spent M9 glucose medium. The fluorescence intensity of each cell at the first time point is set to 100%. Error bars show 95% confidence intervals. (b) Effect of copper chloride on the reporters for the Cpx (pPyebE-gfp) and Rcs (pPrcsA-gfp) systems. Exponential phase M9 glucose cultures were diluted in fresh M9 glucose medium with or without 7 μM CuCl2 and measured at regular intervals by flow cytometry. The fluorescence intensities were normalized to the size of each cell and shown here as the median of at least 36,000 cells. (c) Induction of the CpxR-controlled yebE reporter in the polyacrylamide pad setup by addition of copper. Cells were grown in absence of copper and after 1 h 15 min in the microfluidic device, medium containing 7 μM CuCl2 was perfused. Note that the induction ratio is comparable to the flow cytometry experiment, but that the dynamics are different. Likely, the slower response is related to a delayed and gradually increasing exposure to copper due to diffusion through the polyacrylamide gel. (d) Linearity of the total protein content determinations. The background-corrected integrated band intensities of a number of dilutions of a total protein sample are shown as determined from a silver-stained polyacrylamide gel. (TIF) [file pone.0207181.s001.tif]
